# Sustainable Metal Mixture Separation From E‐Waste Leaching: Flow‐Based System Approach With Closed‐Loop Reutilization of Organic Ligands

**DOI:** 10.1002/cssc.70864

**Published:** 2026-07-07

**Authors:** Vitor A. S. Almodovar, Kevin Moreno, Prashant Ram Jadhao, Kasper Moth‐Poulsen

**Affiliations:** ^1^ Department of Chemical Engineering Universitat Politècnica de Catalunya EEBE Barcelona Spain; ^2^ The Institute of Materials Science of Barcelona ICMAB‐CSIC Barcelona Spain; ^3^ Department of Chemistry and Chemical Engineering Chalmers University of Technology Gothenburg Sweden; ^4^ Catalan Institution for Research & Advanced Studies ICREA Barcelona Spain

**Keywords:** copper, flow chemistry, green chemistry, metal recovery, silver, sustainability

## Abstract

As the amount of electronic waste (e‐waste) continues to grow at an unprecedented rate, the development of efficient and sustainable metal‐recycling methods has become an urgent priority for achieving a greener world. This study reports a flow‐based liquid–liquid extraction system for the selective separation of copper from copper–silver leachates obtained from e‐waste provided using batch leaching with methanesulfonic acid (MSA), a biodegradable and nonoxidizing acid. The selective extraction of copper was achieved using a phenolic oxime, owing to its high affinity for Cu(II) ions. To adjust the optimal conditions, we have screened the effects of different flow rates and residence times on extraction efficiency and also the different metal‐to‐ligand ratios. Optimal conditions were obtained using a metal‐to‐ligand ratio of 1:3 and a flow rate of 0.4 ml^−1^, resulting in a maximum extraction efficiency of 97%. To enhance process sustainability and reduce solvent consumption, a ligand/solvent regeneration step was integrated, enabling copper back‐extraction, recycling of the organic extractant, and implementation of a closed‐loop process. Accordingly, this work presents an approach designed to improve the sustainability of e‐waste recycling through selective metal recovery and closed‐loop reutilization of the ion‐selective ligand.

## Graphical abstract

This paper reports a flow‐based liquid–liquid extraction system for the selective separation of copper from copper–silver leachates obtained from e‐waste. This process enhances sustainability and reduces solvent consumption. In addition, a ligand/solvent regeneration step is described, enabling copper back‐extraction, recycling of the organic extractant, and implementation of a closed‐loop process.

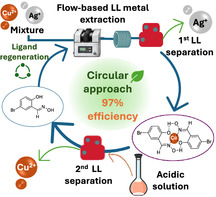

## 1 Introduction

**FIGURE 1:**
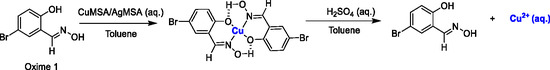
Mechanism of extraction of copper ions from an aqueous CuMSA/AgMSA solution using Oxime 1 as an ion‐selective ligand and stripping of copper ions from the Cu–Oxime complex.

Electronic waste (e‐waste) has become the fastest‐growing solid waste stream worldwide, driven by rapidly increasing demand and shortened product life cycles. According to the United Nations, the yearly production of e‐waste is expected to rise to 82 million tons by 2030 [1]. In fact, in 2022, despite 62 million tonnes (Mt) of e‐waste being generated, only 22.3 wt% were collected and recycled, resulting in the loss of significant quantities of valuable metals and other recyclable materials [1]. The rapid pace of technological advances and increasingly shorter product lifetimes has created an urgent need for environmentally friendly recovery processes. E‐waste contains a wide variety of valuable metals, such as copper, silver, gold, palladium, and rare earth elements, the recovery of which can significantly reduce dependence on primary mining and minimize environmental pollution associated with improper disposal [2, 3]. During the metal recovery process, selective removal of metal ions from leach solutions is crucial for optimizing the overall process. Among the various hydrometallurgical separation methods, liquid–liquid extraction (LLE) is widely used for metal extraction due to its broad scope, ease of implementation, and ability to provide high metal selectivity. The mechanism involved in LLE is the transfer of solute from a leaching solution to an organic solvent. Depending on the ions present in the leaching solutions, different extractants can be employed namely anionic, cationic, and solvating‐type extractants, which present high selectivity for the target metal [4, 5]. Conventional separation techniques, such as chemical precipitation, adsorption, and ion exchange, often suffer from low selectivity, high reagent consumption, or complex downstream processing when applied to multi‐metal e‐waste leachates [6, 7]. At an industrial scale, the extraction process is usually carried out in large mixer‐settlers or columns [8]. Lab‐scale investigations require compact and flexible platforms that allow precise control of residence time, phase ratios, and interfacial area using minimal reagent volumes. Some previous reports have described the microfluidic separation of metal ions through solvent extraction [9, 10, 11]. However, the majority of these reports have addressed the first stage of the separation only, with a particular exception for the work developed by Harvie et al. [9] where they developed a “homemade” system for efficient extraction of metal ions (copper and nickel) using a two‐stage flow reactor. Inspired by Harvie's work, we present a new flow‐based system that applies LLE principles in two stages for the efficient separation of metal ions from a leaching solution containing Cu and Ag ions leached with methanesulfonic acid (MSA) by using peristaltic pumps and advanced membrane liquid–liquid separators. MSA is a biodegradable, nonoxidizing acid that has gained attention as a greener alternative to conventional mineral acids in metal leaching [12, 13]. It is being essentially applied in copper and silver recovery using similar conditions [14, 15]. As a proof‐of‐concept study, we show the selective extraction of copper ions from copper–silver mixtures using phenolic oximes (POs), which exhibit high affinity toward Cu(II) ions (Figure 1) [16, 17]. The mixture containing AgMSA and CuMSA was obtained from metal leaching from e‐waste provided by a printed electronics company (see Supporting Information). Additionally, the system includes a continuous step for recovering the organic ligand in a back‐extraction process, allowing for continuous separations in uninterrupted cycles, thus reducing reagent consumption. This design not only enhances process sustainability but also provides a platform for future continuous and automated separation studies.

## 2 Results and Discussion

**FIGURE 2:**
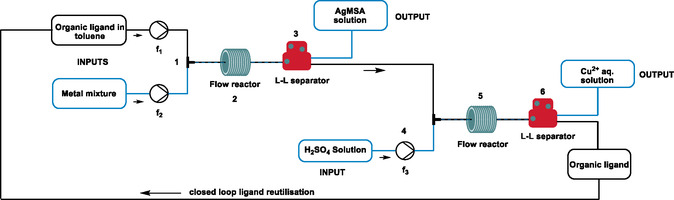
General Scheme for the flow‐based system for metal extraction. **1.** Pump f_1_ introduces the organic ligand solution. Pump f_2_ introduces the aqueous metal mixture (AgMSA + CuMSA). **2**. Both streams mix in the first 10 mL tubing reactor at room temperature, where Cu extraction occurs. **3.** The first phase separator divides the flow into an aqueous AgMSA phase (collected) and an organic phase containing extracted Cu^2+^. **4.** Pump f_3_ then adds H_2_SO_4_ (2.5 M) to the organic phase. **5.** This mixture passes through a second 10 mL tube reactor for Cu stripping. **6**. After the second separator, the aqueous Cu^2+^ solution is collected, and the organic ligand is recycled back to the start.

We present a two‐stage extraction procedure (Figure 2) which extends the concept developed by Harvie et al. [9] to the selective separation of copper and silver from e‐waste‐derived streams and includes an evaluation of ligand recyclability over multiple extraction cycles. In this proof‐of‐concept system, we have two input streams: (i) an organic ligand (dissolved in toluene) and (ii) an aqueous mixture of metal salts (CuMSA and AgMSA) obtained from a batch leaching process (see SI) instead of commercial metal salts as described by Harvie et al. [9]. The two solutions were introduced into the flow‐chemistry separation pathway at controlled flow rates using peristaltic pumps. In the first flow reactor, Cu ions react with the organic ligand to form Cu–ligand complexes. The formation of this complex was confirmed previously by performing a small batch solvent extraction and analyzing the resulting organic phase by UV–vis spectroscopy (Figure S2). The presence of an absorption band centered at 357 nm is consistent with the formation of a Cu–oxime complex. The biphasic mixture then passes through a liquid–liquid separator, yielding an aqueous AgMSA solution as the first output. Subsequently, the organic phase, which contains the Cu complex, enters the second stage of the flow system. Simultaneously, a solution of H_2_SO_4_ is introduced as a third input stream and mixed with the organic phase. These two solutions remain in contact for a defined time in a flow reactor, promoting the stripping of the metal from the complex. The resulting biphasic organic‐aqueous solution then passes through a second liquid–liquid separator, promoting the production of *two outputs*: a CuSO_4_ aqueous solution and the organic solution containing the pure ligand, which is recycled in a closed loop (Figure 2). The dimension of the system was chosen to be practical in a small lab‐scale demonstration. However, based on the principles presented here, we envision the scale‐up of the process.

### 2.1 Flow Rate Optimization

**FIGURE 3:**
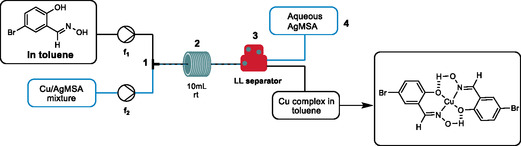
Optimization of the flow rates and residence times for the first section of the system.

The optimization of the flow rates and residence time was first carried out using water and toluene in the absence of metal ions or extractant. This step allows us to guarantee stable and reproducible flow conditions within the extraction system. Initially, the focus was on the first stage of the system, in which the target metal ion was transferred from the aqueous feed solution to the organic extractant phase (Figure 3). After phase separation, the aqueous stream was collected and then analyzed off‐line using inductively coupled plasma mass spectrometry (ICP‐MS; see Table 1). This step allows us a direct evaluation of extraction efficiency. At this stage, the effect of flow rate on copper extraction was investigated using flow rates in the range of 0.4–1 mL/min, corresponding to residence times of 13.85–5.54 min, matching the operational limits of the flow system, while maintaining a fixed metal‐to‐ligand ratio of 1:2. Starting with a flow rate of 400 μL/min and a corresponding residence time of 13.85 min, copper extraction reached 91%, indicating high efficiency in metal separation using phenolic oxime with selectivity for copper ions. It was verified that increasing the flow rate and reducing the residence time slightly reduced the efficiency, resulting from less time for coordination between the Cu ions and the ligand under flow dynamic conditions. A negligible loss of Ag ions was also verified in the process, confirming the selectivity of the ligand for Cu ions and highlighting the advantage of this system for selective copper recovery from mixed‐metal e‐waste leachates. Table 1 summarizes the results of the ICP analysis of the samples collected at various flow rates.

**TABLE 1:** Effect of flow rate and residence time on Cu(II) extraction from mixed Cu–Ag Methanesulfonate leachates using phenolic oxime extractant.

Flow rate (*f*_1_ and *f*_2_), mL.min^−1^	Residence time, min	[Ag], ppm	[Cu], ppm	Cu extracted, %
0.4	13.9	104,7	5,0	91
0.6	9.2	104,8	9,5	83.5
0.8	6.9	103,9	11,9	78.3
1	5.5	104,9	12,1	77.8

*Note:* Initial metal concentrations in the aqueous feed:[Cu]_0_ = 54.5 ppm and [Ag]_0_ = 130,3 ppm as determined by ICP‐MS before extraction. Metal‐to‐ligand ratio = 1:2.

### 2.2 Metal‐to‐Ligand Stoichiometric Ratio Optimization

To further optimize metal extraction, the metal‐to‐ligand molar ratio was investigated, fixing the flow rate at 400 μL/min, which corresponded to the longest residence time evaluated (13.9 min). In addition to the ratio tested for flow rate optimization, two more ratios were evaluated. Table 2 summarizes the effect of the ratio on the copper extraction efficiency. When the ratio was adjusted to 1:1, the efficiency decreased to 87%, showing that an equimolar proportion of ligand is not enough to achieve the full complexation of the Cu ions under continuous flow conditions. The relatively high residual concentration of copper ions in the aqueous phase suggests incomplete coordination due to kinetic limitations and restricted ligand availability within the residence time of the system. In addition, a silver loss of 8% was observed. As explained before, using a 1:2 metal‐to‐ligand ratio improved copper extraction to 91%, accompanied by a decrease in residual copper concentration to 5.00 ppm. However, a high apparent silver loss was observed under these conditions. This silver loss is probably related to phase carryover or transient interfacial retention rather than coordination extraction issues, given the known low affinity of phenolic oximes for Ag(I) ions [8]. This emphasizes the significance of hydrodynamic optimization and phase disengagement efficiency. Increasing the ratio to 1:3 resulted in a maximum efficiency of 97% with a residual aqueous copper concentration of 1.60 ppm. It is important to highlight that Ag loss under these conditions decreased significantly to 3.6%, indicating that excess ligand not only enhances Cu complexation but also stabilizes phase behavior and improves selectivity under flow conditions. The presence of an excess of ligand compensates for some possible kinetic constraints associated with flow‐based systems, where the equilibrium may not be fully established. These results show that the optimized metal‐to‐ligand ratio is a critical parameter for maximizing the extraction efficiency in LLEs. Working in a closed loop, the Ag “loss” is probably due to lower efficiency in the phase separation rather than an irreversible loss. Such losses can be minimized through process optimization and improved phase disengagement.

**TABLE 2:** Cu extraction at 0.4 mL.min^−1^ using different metal‐to‐ligand ratios.

Metal: Ligand	[Ag], ppm	[Cu], ppm	Cu extracted, %	Ag loss, %
1:1	119, 8	7, 7	87	8
1:2	104, 8	5, 0	91	20, 4
1:3	125, 7	1, 3	97	3,6

*Note:* Initial metal concentrations in the aqueous feed: [Cu]_0_ = 54.5 ppm and [Ag]_0_ = 130, 3 ppm as determined by ICP‐MS before extraction.

### 2.3 Ligand Regeneration, Cyclability and Cu Recovery Efficiency

Based on the results obtained for the selective copper extraction in the first stage, an additional module was assembled to enable ligand recyclability and the production of Cu salts. A single continuous workflow was performed without independent or disconnected steps. The 2^nd^ step was designed to strip Cu(II) ions from the loaded organic phase, regenerating the oxime for subsequent extraction cycles. In the 2^nd^ step, the organic phase exiting the first liquid–liquid separator enters directly into a second flow reactor, where it meets an aqueous H_2_SO_4_ (2.5 M) solution under controlled flow conditions (400 µL/min). During this period of contact, the ligand is regenerated, and there is the formation of CuSO_4_, which remains in the aqueous phase. The mixture is passed through a second liquid–liquid separator, and the aqueous solution containing CuSO_4_ and the organic solution containing the regenerated organic ligand are collected. The final concentration of Cu ions was analyzed offline using ICP‐MS, and the structure of the regenerated ligand was confirmed by NMR to evaluate its use in another extraction cycle. A final concentration of 42.3 ppm Cu ions was obtained in the aqueous solution with no presence of Ag, demonstrating effective copper recovery and excellent selectivity. This corresponds to an overall efficiency of 77.6% for the complete closed‐loop system. The observed decrease in efficiency can be attributed to the cumulative effect of multiple steps within the integrated system, to the kinetic limitations imposed by the continuous‐flow nature of the system, and to the possible incomplete stripping from the ligand in the last stage. In addition, the absence of Ag(I) in the recovered aqueous phase confirmed that the decrease in efficiency was not associated with the loss of selectivity. The spectra confirmed that the ligand structure remained intact, indicating its suitability for subsequent separation cycles. The introduction of this regeneration step is crucial for assessing the practical viability of the system in terms of sustainability, as it reduces the reagent consumption and waste generation compared to conventional batch extraction processes.

After the regeneration, the ligand was tested through 5 extraction cycles. ICP‐MS was used to analyze samples collected after the third and fifth cycles to assess the stability and extraction performance of the ligand during the recycling process. The results demonstrated that the copper concentration detected after the first extraction stage was residual (Table 3). Notably, the results (values below 0.1 ppm) suggest an improvement in extraction performance in the first extraction, likely due to the stabilization and optimization of the system.

**TABLE 3:** Cu extraction at 0.4 mL⋅min^−^^1^ through 5 cycles using recycled ligand.

Cycle	[Cu], ppm	Cu extracted, %
1	1, 3	97
3	<0, 1^a^	>97
5	<0, 1^a^	>97

*Note:* Initial metal concentrations in the aqueous feed: [Cu]_0_ = 54.5 ppm as determined by ICP‐MS before extraction.

a Values below the quantification limit of the ICP–MS method.

### 2.4 Methanesulfonic Acid as Stripping Agent

In order to replace sulfuric acid with a more environmentally friendly acid, an extraction cycle was performed using MSA as a stripping agent. An overall efficiency of 79.7% was observed to compared with the 77.6% obtained with sulfuric acid. Although sulfuric acid was selected for the described process, the preliminary results with methanesulfonic acid highlight the potential for further improving the sustainability of the overall process.

### 2.5 2‐MeTHF as an Alternative to Toluene

To evaluate the use of greener solvents, 2‐methyltetrahydrofuran (2‐MeTHF) was used in place of toluene in the first stage of the process. While 2‐MeTHF is an attractive green solvent, its use resulted in significantly reduced copper extraction efficiency and unexpected silver losses under the conditions investigated. Owing to these unfavorable performance metrics, 2‐MeTHF was not pursued further; full experimental details and results are provided in the Supporting Information.

## 3 Conclusion

This study demonstrates the design of an efficient flow system for metal separation. This system provides proof of concept for the use of flow‐based methods, including continuous flow‐based membrane separation systems, for the LLE of metals from e‐waste. Selective extraction of Cu(II) from Cu/Ag methanesulfonate leachates was achieved with high selectivity using phenolic oxime extractants, while negligible silver co‐extraction was observed under optimized conditions. The integration of ligand and solvent regeneration allows continuous closed‐loop recycling of the organic phase, reducing reagent consumption and waste generation. The ligand was successfully recycled over five consecutive extraction cycles without a decrease in extraction efficiency. This flow‐based approach represents a sustainable and scalable platform for metal recovery from e‐waste. The closed‐loop modular design opens new opportunities for automated and continuous metal separation studies with clear potential for scale‐up and integration into industrial recycling workflows.

## 4 Experimental Section

### 4.1 General Methodology

In this proof‐of‐concept system, we have two input streams, an organic ligand (organic phase in toluene) and a mixture of metal salts (CuMSA and AgMSA) obtained from a leaching process. These two solutions enter a flow chemistry separation path. In the first flow reactor, the Cu ions react with the organic ligand to form Cu–ligand complexes. After passing through a liquid–liquid separator is possible to obtain a pure AgMSA solution as the first output. The organic phase, containing the Cu complex, then enters a new flow stream. Simultaneously, a solution of H_2_SO_4_ (2.5 M) is pumped as a third input stream and mixed with the organic phase. These two solutions remain in contact for a defined time in a flow reactor, promoting the stripping of the metal from the complex. The resulting biphasic organic‐aqueous solution then passes through a second liquid–liquid separator, promoting the production of two outputs: a CuSO_4_ aqueous solution and the organic solution containing the pure ligand, which is recycled back to the start.

Before each experimental run, the flow system was sequentially flushed with water and toluene to remove traces of leftover metal salts to be used.

### 4.2 Materials

The waste electronics were provided by MelsenTech. The extractant ligand, 5‐Bromo‐2‐hydroxybenzaldehyde, was purchased from Fisher S.L. The metal salts were obtained following the procedure described in the Supporting Information.

#### 4.2.1 Flow System Setup

Two membrane liquid–liquid separators were purchased from Zaiput Flow Technologies. Vapourtec R2S+ module based on acid‐resistant V‐3 peristaltic pumps. SF‐10 peristaltic pump from Vapourtec. Vapourtec Standard PFA tubing with an inner diameter of 1 mm and either 1* = *4″−28 PFA nuts and adaptors were used for the connections to the reactors and pumps. Two PFA tube reactors (inner diameter/internal bore of 1 mm, 10 mL, operative T: ambient to 150°C) from Vapourtec were used for the LLE reactor.

## Funding

This study was supported by the HORIZON EUROPE Framework Programme (101070556).

## Conflicts of Interest

The authors declare no conflicts of interest.

## Supporting information

Supplementary Material

## Data Availability

The data that supports the findings of this study are available in the Supporting lnformation of this article.
