# Supplementary material for: Sustainable Metal Mixture Separation From E‐Waste Leaching: Flow‐Based System Approach With Closed‐Loop Reutilization of Organic Ligands
Source: ChemSusChem. 2026 Jul 7;19(14):e70864. doi: 10.1002/cssc.70864 (PMC13342483; doi:10.1002/cssc.70864)
Supplement: Supplementary file 1 — Supplementary Material [file CSSC-19-e70864-s001.pdf]

Supporting Information

Sustainable metal mixture separation from e-waste leaching: Flow-based system approach

Vitor A. S Almodovar[a], Kevin Moreno[a], Prashant Ram Jadhao[a], Kasper Moth-Poulsen[a]*

[a] Dr. Vitor A. S. Almodovar, Dr. Kevin Moreno, Dr. Prashant Ram Jadhao, Dr. Kasper Moth-Poulsen
Department of Chemical Engineering Universitat Politècnica de Catalunya, EEBE, Eduard Maristany 10–14, 08019 Barcelona (Spain)
E-mail: kasper.moth-poulsen@upc.edu

1. General procedure for batch copper and silver leaching

The recycling of metals from wasted printed electronics was performed using methane sulfonic acid (MSA) in the presence of hydrogen peroxide, which acts as an oxidant. The samples containing Cu and Silver were shredded to a size of 2 x 2 cm (Figure S1(a) and S1(b)) and further used for experimental work. Based on the study, 100% recycling of Cu as a metal precursor (CuMSA) was achieved at the conditions mentioned in Table S1.

Table S1. Reaction conditions for the recycling of Cu

**MSA Conc. (M)**	**H_2O_2 Conc. (M)**	**Stirring Speed (rpm)**	**S/L Ratio (g/mL)**	**Time (Min)**	**Temperature (°C)**
0.6	0.4	400	1:20	30	40

Around 98% recycling of silver was achieved at the conditions mentioned in Table S2.

Table S2. Reaction conditions for the recycling of Ag

**MSA Concentration (M)**	**H_2O_2 Concentration (M)**	**Stirring Speed (rpm)**	**S/L Ratio (g/mL)**	**Time (Min)**	**Temperature (°C)**
8	3	300	1:20	30	60

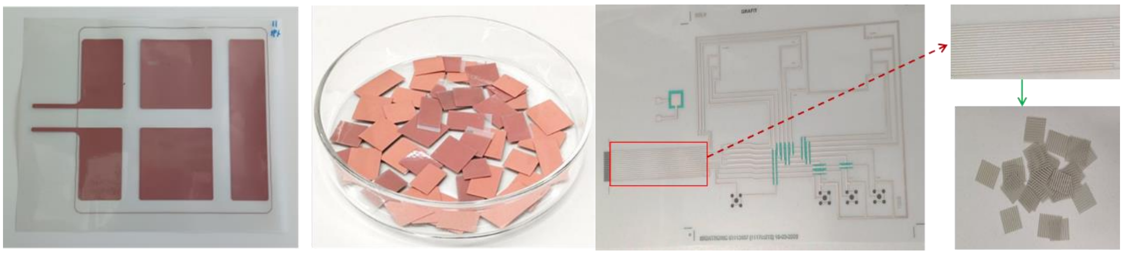

a b

Figure S1. PE waste containing Cu (a) and PE waste containing Silver (b)

2. Uv-vis spectra

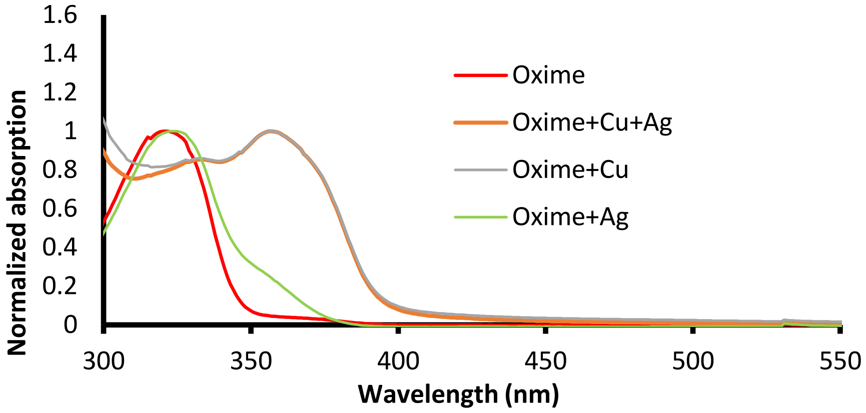

Figure S2. UV–Vis spectra of oxime and its complexes with Cu, and Ag during the extraction process.

Table S3. Maximum absorption wavelengths (λ_{max}) of 5-bromo-3-hydroxybenzaldehyde oxime and its Cu and Ag complexes in toluene.

**Sample in Toluene**	**λ_{max} (nm)**
**5-Br-3-hydroxybenzaldehyde oxime**	321
**Cu oxime complex**	357
**Ag oxime complex**	323

3. 2-MeTHF as an alternative to Toluene

To evaluate the use of greener solvents, a run using 2-MeTHF as an alternative to toluene was performed. 2-MeTHF is a promising green solvent that can replace THF because of its low water solubility, enhanced phase separation, reduced carbon emissions, and improved stability. We repeated the first stage of the process, starting with an initial aqueous feed containing 130.33 ppm Ag(I) and 54.46 ppm Cu(II). The flow rate was fixed at 400 µl/min, and the metal-to-ligand ratio was 1:3. After collection in the first stage, the copper and silver concentrations were determined offline using ICP-MS. It was verified that the copper efficiency decreased markedly to 22.4%. Additionally, a substantial decrease in the aqueous Ag(I) concentration was observed (56.9%). According to the well-established lack of chemical affinity between phenolic oximes and Ag(I), this behaviour cannot be attributed to coordination extraction. Although 2-MeTHF is often described as having low water miscibility, its behaviour is slightly different from that of completely non-miscible solvents such as toluene. This partial miscibility and distinctive phase equilibrium have been documented in ternary LLE systems, and can result in more complex phase separation dynamics during continuous liquid–liquid extraction.[17,18]

4. ^{1}H NMR of the ligand before and after an extraction cycle

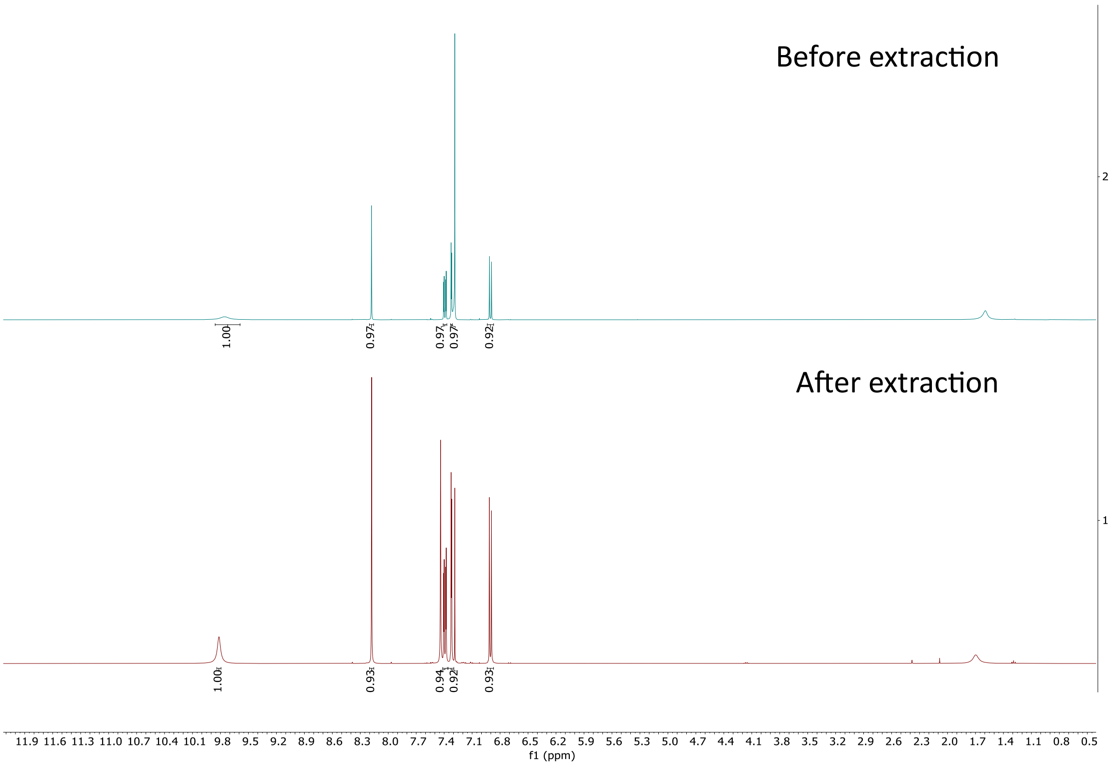

Figure S3. ^{1}H NMR of the extractant before and after metal extraction.
